# Photostability of Topical Agents Applied to the Skin: A Review

**DOI:** 10.3390/pharmaceutics12010010

**Published:** 2019-12-20

**Authors:** Agata Kryczyk-Poprawa, Anna Kwiecień, Włodzimierz Opoka

**Affiliations:** Department of Inorganic and Analytical Chemistry, Jagiellonian University Medical College, Faculty of Pharmacy, 9 Medyczna Street, 30-688 Krakow, Poland; anna.kwiecien@uj.edu.pl (A.K.); wlodzimierz.opoka@uj.edu.pl (W.O.)

**Keywords:** photostability, photodegradation, glucocorticosteroids, retinoids, antifungal drugs

## Abstract

Topical treatment modalities have multiple advantages starting with the convenient application and non-invasive treatment and ending with the reduction of the risk of the systemic side effects. Active pharmaceutical substances must reach the desired concentration at the target site in order to produce a particular therapeutic effect. In contrast to other dosage forms topical agents applied to the skin may also be susceptible to photodegradation after application. That is why the knowledge of the susceptibility of these topical drugs to UV irradiation, which may contribute to their degradation or changes in chemical structure, is very important. Active pharmaceutical substances used in dermatology may differ both in chemical structure and photostability. Furthermore, various factors—such as light intensity and wavelength, pH, temperature, concentration—can influence the photodegradation process, which is reflected in particular in kinetics of photodegradation of active pharmaceutical substances as well as both the quantitative and qualitative composition of by-products. The aim of this study was to conduct a systematic review of the photostability of dermatological drugs, as well as of other substances commonly applied topically. The photostability of glucocorticosteroids, retinoids, and antifungal drugs as well as non-steroidal anti-inflammatory drugs applied topically and selected UV-filters have been discussed. Furthermore, the impact of photoinstability on the effectiveness of pharmacotherapy and some photostabilization strategies have been also included.

## 1. Introduction

Skin is an important administration route of drugs for both topical and systemic therapy. Of all available routes of drug administration, the topical application has some advantages. Administration of the drug to the skin for systemic effect (1) bypasses the effect of the first pass through the liver, (2) is an easy and patient-friendly route of administration, (3) allows some control of the delivery of the drug. In recent years, many studies have focused on the application of the drug to the intact and healthy skin as an alternative to other routes of administration to achieve systemic effects. Transdermal or dermal systems account for approximately 40% of total drug delivery product candidates at the stage of clinical trials [1,2]. On the other hand, to achieve local effect, the epicutaneous or topical routes of administration are preferred options [3]. As regards local effect, topical administration facilitates the safe use of the drug, and reduces the chances of adverse reactions [2,3]. Topical agents applied locally to the skin are used in the treatment of skin diseases (e.g., antifungal drugs, antiseptic drugs, ultraviolet (UV) radiation-blocking agents, anti-inflammatory, or anti-allergic drugs) or could penetrate deeper into local tissues, e.g., drugs used for relieving pain and reducing inflammation of muscles and joints. On the other hand, it should be stressed that following topical administration the active pharmaceutical ingredient (API) must reach the desired concentration at the target site so that it provides a particular therapeutic effect. Appropriate API concentration will be obtained after the application of the recommended amount of drug product (a finished dosage form) to the skin [4]. The skin is the largest organ of the human body (1.5–2.0 m^2^) that protects against external environmental factors, and thus is exposed to, among others, UV radiation and xenobiotics. Overcoming the barrier that constitutes the skin is possible for lipophilic substances with log P in the range of 1–3 and those with a low molecular weight (MW < 500 Da) [2]. Therefore, many substances applied topically that penetrate through the stratum corneum, reach the dermis and via dermal microcirculation could get into the systemic circulation causing systemic effects [2,3,5].

The stability studies of APIs and drug products are a priority in the research and development of drugs. Information about the stability of the drug is necessary to ensure its appropriate quality, effectiveness, and safety for the patients [6,7]. General rules for conducting stability tests are included in the Guidance for Industry Q1A(R2) Stability Testing of New Drug Substances and Products which was developed by International Conference on Harmonisation of Technical Requirements for Registration of Pharmaceuticals for Human Use [8]. The ultimate goal of the stability studies is to provide industry guidance for ensuring the stability of API during all stages of production, packaging and storage. The test results provide a basis for determining the durability of a drug product to ensure that the medicine is stored properly under the critical environmental conditions (light, humidity, and temperature) and will remain in full effect until the end of its useful life. It is commonly known that UV irradiation could contribute to the degradation of drug substances and drug products, which could have potential consequences in the decrease or loss of their pharmacological activity and thereby have an effect on the effectiveness and safety. The crucial issue related to the stress testing studies is the photostability testing of new drug substances and products which is included in ICH Q1B [9]. All APIs are protected from radiation during storage but during application to the skin, in case of exposure to the sun, photodegradation can occur. In addition a very thin layer (about 1 mm) of the drug products are often applied directly to the skin. Therefore, it is necessary to specify the photostability of API alone, as well as in the presence of other substances, including excipients or cosmetic ingredients [10].

Ultraviolet radiation (UVR) is divided into the following bands: UVA (315–400 nm), UVB (280–315 nm), and UVC (100–280 nm). All UVC and 95% of UVB radiation are absorbed by the protective ozone layer in the stratosphere. UVA and only about 5% of UVB radiation reach the Earth’s surface. UVC radiation with the highest energy is strongly mutagenic, but only reaches the epidermis; and 90% of UVB radiation is retained by the stratum corneum and is responsible for the skin erythema and immunosuppression. Nucleic acids are the main chromophores for UVB radiation (maximum absorption for both DNA and RNA is about 260 nm), that is why there are many mutations, mainly UVB fingerprint mutations. However, recent studies have implicated an increase in the role of UVA as a carcinogen [11,12,13]. UVA radiation is the most important from the point of view of everyday life, because it is responsible for the majority of phototoxic and photoallergic reactions, telangiectasia, mutagenic, and carcinogenic effects. Research carried out by Huang et al. showed that UVA radiation induces mutations in the epidermal basal membrane, where keratinocytes are dividing, which gives rise to the skin tumors [14]. It reaches the Earth’s surface with relatively equal intensity throughout the year, penetrates through the glass, and additionally penetrates deeper into the skin compared to UVB radiation, which intensifies its harmful effects. Repeated exposure causes mutations in the p53 gene [15]. Depending on the intensity and wavelength, UV irradiation could also affect the stability of the drug. According to the guidelines contained in Q1B ICH, the following radiation sources are recommended for the photostability tests: (i) xenon or metal-halogen lamp that creates artificial daylight, combining the range of visible light and UV with emission standard D65/ID65 (according to ISO 10977); (ii) simultaneous use of a fluorescent lamp with cold white light with a power similar to external daylight according to ISO 10977 and a fluorescent lamp close to UV with a range of 320 nm to 400 nm and a maximum emission energy between 250 nm and 270 nm. However, interior lighting sources have changed with the adoption of light emitting diodes (LEDs) which may increase the risk of photodegradation during the out-of-package in-use period [9,16]. On the other hand, several papers discussed the lack of photostability testing of pharmaceutical drug substances during or after administration. It is therefore necessary to examine how the existing instruments and existing guidelines aid the understanding of the photostability of topical drugs, which could be exposed to a significant amount of light after application to the skin [10,17,18,19,20,21].

**Figure 1:**
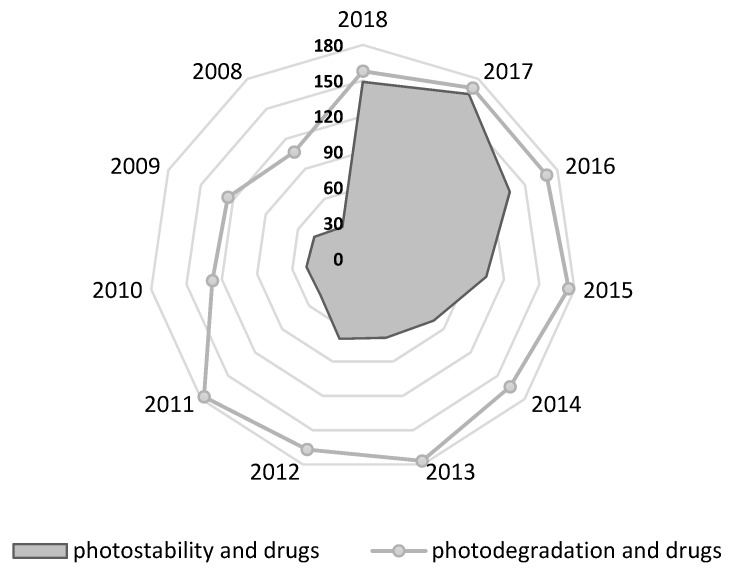
Results by year based on the search: “photostability and drugs” and “photodegradation and drugs” (2008–2018, PubMed).

**Figure 2:**
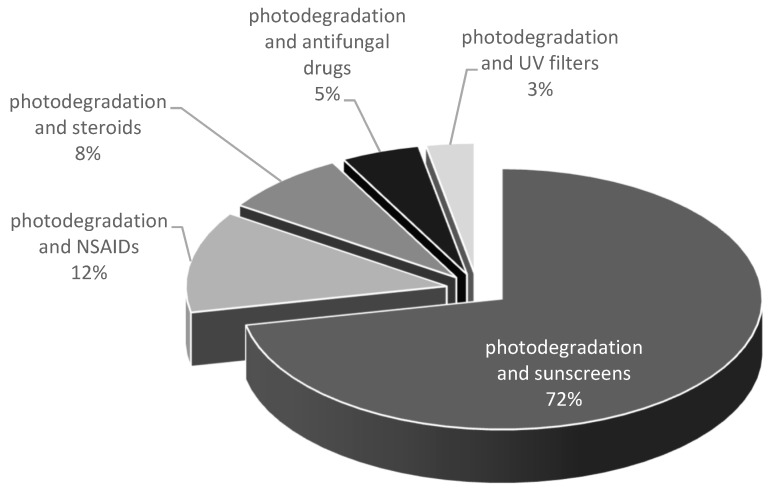
Results based on the search for the photodegradation of the main classes of drugs reported in PubMed.

Two main categories of drug products applied topically to the skin are identified: products applied for local action (creams, gels, sprays, solutions) and products applied for systemic effects—transdermal drug delivery systems. The aim of this study was to conduct a systematic review of the photostability of dermatological drugs applied topically to the skin for local effect. The photostability of the most important groups of topical drugs used in dermatology—such as glucocorticosteroids, retinoids, and antifungal drugs—are discussed. In addition, the photostability of nonsteroidal anti-inflammatory drugs (NSAIDs) applied topically as well as selected UV-filters is also presented. The US Food and Drug Administration FDA considers sunscreen products to be OTC drugs. In Europe, however, they are considered cosmetics. The European Union, Australia, and Japan allow several UV filters that are not available in the United States. The FDA list contains only 16 permitted radiation protective substances, 11 of them are approved in Europe, however, they differ in the values of the maximum concentrations. In Europe, Annex VI to the EU Cosmetics Regulation contains 29 substances, 2 of which are physical filters [22,23]. The stability of selected chemical UV filters and potential interactions of physical UV filters with other compounds under UV irradiation are discussed. The preliminary overview of the current literature in PubMed based on search: photostability and drugs identified 1303 results, including 993 in the last 10 years; photodegradation and drugs identified 3392 results, including 1596 in the last 10 years. The results by year are presented in Figure 1. The percentage of main classes of drugs in the total amount of the photodegradation search are presented in Figure 2. The literature search was narrowed down to the particular groups of drugs listed above. The most important groups of drugs described in the article and examples of APIs most relevant from the point of view of photostability are presented in the Table 1. Furthermore, the impact of photoinstability on the safety and effectiveness of pharmacotherapy has been also included. To the best of our knowledge, for the first time, an attempt was made to systematize the data related to the photostability of drugs applied topically to the skin.

## 2. Classes of Drugs

### 2.1. Topical Glucocorticosteroids

Topical glucocorticosteroids are among the most effective and most commonly used drugs for skin diseases. In 1952, Sulzberger and Witten applied for the first time hydrocortisone topically for the treatment of skin diseases. Pharmacological action of topical glucocorticosteroids takes place through a specific receptor. Glucocorticosteroids bind to cytoplasmic receptors that transport the drug to the cell nucleus, where the complex modifies gene transcription. In the nucleus itself there are also receptors for the glucocorticosteroids. Then, there is the attachment of regulatory DNA sequences, called glucocorticosteroid acting elements (GRE, glucocorticoid response elements), which are contained in the regulatory part of the genes coding proteins synthesized in the cell response to glucocorticosteroids. These receptors are found both in the epidermis and dermis. Topical glucocorticosteroids have anti-inflammatory, anti-proliferative, and immunosuppressive effects. The topical side effect is primarily the thinning of the epidermis and dermis [80,81]. Drugs from this group are classified according to the strength of topical action in seven groups. While creating this classification, the physicochemical properties of these drugs, the concentration of the API and its affinity for specific receptors were also taken into account. Betamethasone dipropionate belongs to the group of the strongest topical glucocorticosteroids, whereas betamethasone valerate is classified as a glucocorticosteroid with high potency [82].

The studies on the photostability of glucocorticosteroids laid the foundations for the development of modern organic photochemistry [83]. Cross-conjugated glucocorticosteroids—such as prednisolone, betamethasone, and triamcinolone—are highly unstable. It has been shown that general patterns in the decomposition of corticosteroids under UV irradiation involve rearrangement of cyclohexadienone moiety, resulting in two main photoproducts: ’lumiderivatives’ and ‘photolumiderivatives’, and as a consequence of side chain loss causing the formation of ‘androderivatives’ [24,25]. In case of betamethasone esters, the photoinstability resulted in generation of photodegradation products, which proved to be toxic/phototoxic [26]. The photostability of the hydrophobic betamethasone ester, which is easily transported through the skin—betamethasone-17 valerate (9-floro-11β,21-dihydroxy-16β-methyl-3,20-dioxopregna-1,4-dien-17-yl pentanoate) has been thoroughly investigated. This synthetic glucocorticosteroid is highly photolabile. The photodegradation rate was dependent on solvent dielectric constant, ionic strength, buffer concentration, and ingredients used in cream and gel formulations [27]. The lower photostability of betamethasone-17 valerate was observed in the gel formulation compared to that for the cream formulation. The difference in the composition of the formulations (cream: betamethasone-17 valerate 0.1, carbomer (940) 1.5, propylene glycol 8.0, cetostearyl alcohol 7.0, isopropyl alcohol 2.0, ethyl paraben 0.2, deionized water 81.0 and gel: betamethasone-17 valerate 0.1, carbomer (940) 0.7, hydroxyethyl cellulose 0.5, propylene glycol 20.0, diisopropanolamine 0.5, isopropyl alcohol 2.0, ethyl paraben 0.2, deionized water 75.9% (*w*/*w*) has an impact on the photostability of API. Further research on the photostabilization of betamethasone-17 valerate in cream and gel formulations, through the use of titanium dioxide, vanillin, or butyl hydroxytoluene showed promising results [27].

The comparative photolysis of betamethasone and its esters: betamethasone-17 valerate and betamethasone 21-phosphate (disodium salt) under UVB irradiation has been studied in solution and in pharmaceutical dosage forms [25]. Betamethasone 21-phosphate is a water-soluble form of parent corticosteroid. Betamethasone-17-valerate was more stable under experimental conditions. UVB photolysis of betamethasone-17-valerate in commercial cream was less efficient than in methanol solution on exposure to low doses of UVB irradiation (5 J/cm^2^). Furthermore, betamethasone was more stable in methanol than in water, but in both cases the same photoproducts were formed. Chlorocresol used as a preservative in this formulation showed photoprotective effect as it possesses the phenolic chromophore, which absorbed light in the UVB range [25]. Furthermore, optical properties of the dosage form (gel, cream) compared with a solution could also impact on the photostability of APIs. Photodegradation of betamethasone is linked with the decrease of its anti-inflammatory activity what has been shown in the test on THP-1 cells [25]. 

Teng et al. characterized degradation pathways for mometasone furoate. This synthetic glucocorticosteroid revealed the highest stability at pH < 4; increasing pH and decreasing ionic strength decrease the stability of mometasone furoate in aqueous media [28].

The influence of UVB irradiation on the photostability of hydrocortisone 21-acetate in methanol, PBS, solid-state and in a commercial cream was investigated by Caffieri et al. [29]. The photolysis of hydrocortisone 21-acetate in the commercial cream caused 20% decrease of its concentration. The preparation of similar cream with hydrocortisone 21-acetate, but without two parabens (methyl- and propyl p-hydroxybenzoates) resulted in faster photolysis of investigated compound. This suggests a significant photoprotection effect of the preservatives because of the presence of the phenolic chromophore being able to absorb UVB light. A further insight was that the excipients affect not only the rate of photolysis, but also the nature of photoproducts formed under irradiation [29]. 

The solubility of the drug substance in a formulation is also essential for its photostability. APIs could be both dispersed or dissolved in a semisolid dosage form. The differences in the photostability of API in different bases could be the result of different solubilities in these formulations. A possible relationship between solubility and photostability was demonstrated for corticosteroids, e.g., hydrocortisone and triamcinolone acetonide [84]. The addition of pigments, e.g., TiO_2_ or ZnO could stabilize the APIs by reflecting, scattering, and/or absorbing most of the UV-rays [85]. Because of these features, these pigments are used as UV filters in sun creams. TiO_2_ and ZnO have been proven to be useful in photostabilization of photosensitive corticosteroids. The addition of TiO_2_ or ZnO improved photostability of triamcinolone acetonide in basis cream [84].

The experiment carried out by Cacciari et al. showed that the presence of oxygen could have special significance to the rate of the photodegradation of corticosteroids. The photodegradation process of prednisolone under UVB irradiation was based on the two pathways: direct photolysis and self-sensitization via photogenerated reactive oxygen species [30]. The photogeneration of OH^•^, and then, their attack on the corticosteroid could be responsible for the photodegradation process of prednisolone, dexamethasone, triamcinolone 16,17-acetonide or fluocinolone 16,17-acetonide [30,31,86,87]. 

Glucocorticosteroids are also used in the treatment of the scalp diseases. The photostability of pharmaceutical preparations during the usage on the scalp is particularly important. Desonide is a glucocorticosteroid used topically to relieve inflammatory symptoms and pruritus in diseases such as: contact dermatitis, atopic skin inflammation, psoriasis, or lichen planus. The drug was at the forefront of the most commonly prescribed steroids in atopic skin lesions. Santa et al. have reported that this compound is unstable in the commercially-available hair preparation. After 2 h of UVA irradiation, the content of the API decreased below 90%, which at the frequency of application twice a day raises concerns as to the maintenance of the therapeutic concentration [33]. In the context of photoinstability, particularly important is the investigation of Rosa et al. about the stabilizing effect of benzophenone-3 on desonide. The results of studies on the photostability of desonide conducted by Rosa et al. indicate the protective role played by the addition of benzophenone-3 to the formulation. After 15 h of UVA irradiation, there was only 1.49% loss of the active substance, which in comparison to 61% of the loss in the case of a commercial product indicates the contribution of the UV filter to the stabilization of desonide [34]. 

Photodegradation of active substances from the steroid group could be a source of new compounds of unknown structure and activity. Numerous studies have confirmed the phototoxic potential of the topically applied substances and photoproducts formed under UV irradiation. The phototoxic potential of triamcinolone 16,17-acetonide has been investigated in in vitro studies. The drug has undergone extensive photolysis to three primary photoproducts, which were isolated and then subjected to toxicity tests: photohemolysis, linoleic acid peroxidation, protein photodamage, 3T3 photocytotoxicity, and DNA photodamage. The phototoxicity or toxicity were proven for two photodegradation products of triamcinolone 16,17-acetonide, which was connected with the photosensitizing activity of investigated compound. One of the photoproducts, 9a-fluoro-17b-hydroperoxy-11b-hydroxy-16a,17a-(1-methylethylidenedioxy)-androsta-1,4-dien-3-one has been proven to be more toxic and phototoxic than triamcinolone 16,17-acetonide [87]. The phototoxicity of both the fluocinolone 16,17-acetonide and its photoproducts under UVA and UVB irradiation was evaluated. In this case, the most probable mechanism of phototoxicity is connected with radicals forming during photodegradation of parent drug under UVB irradiation, as well as with reactive oxygen species activity primarily under UVA irradiation [31].

Photostability of flumethazone and flucinolone acetonide was assessed by photoactivation in the skin exposed to irradiation so as to determine whether the photodegradation of corticosteroids is directly linked with their ability to cause allergic reactions. For this purpose, Miolo et al. used the pig skin as ex vivo model as well as bovine serum albumin, proteins, peptides, and amino acids to be a source of information on processes occurring in the skin. The photoproducts found in in vitro studies were the same as in the case of pig skin after UVB irradiation except flucinolone acetonide hydroperoxide which indicates the similarity of the processes occurring in the skin upon UVB exposure and the likelihood of photoreactivity of photoproducts in the skin of sun exposed patients [32].

### 2.2. Retinoids

The problem of acne vulgaris, due to its frequent occurrence and difficulties in treatment, is still current. The goal of acne treatment is to reduce the production of sebum, to get rid of micro-nodes and to prevent the formation of new ones, to inhibit the development of *P. acnes* and to reduce inflammation [88,89]. Nowadays, three generations of retinoids are distinguished. The first generation includes natural compounds: retinol, tretinoin, and isotretinoin. The second generation includes monoaromatic synthetic compounds: acitretin and etretinate. The third generation are synthetic polyaromatic derivatives, which include adapalene, tazarotene, and bexarotene. Difficulties in assigning adapalene to a generation are caused by its different structure (a derivative of 1-naphthalenecarboxylic acid). However, based on its similar mechanism of action, adapalene is also included in the third generation retinoids [90]. In the treatment of acne vulgaris, topical retinoids (isotretinoin, adapalene, tazarotene) are used as monotherapy and in complex products, e.g., adapalene in combination with benzoyl peroxide. Tazarotene has been approved for the treatment of acne vulgaris only in the US, while in Europe it is used off label. The only retinoid used orally to treat severe forms of acne vulgaris and rosacea is isotretinoin. Retinoids are a group of substances of vitamin A activity. In 1982, isotretinoin (a tretinoin stereoisomer) was approved for use, which was a breakthrough in the treatment of acne. Retinoids act by activating receptors located in the cell nucleus, which leads to the expression of appropriate genes. Two families of receptors are distinguished: RAR-receptors for retinoic acid and RXR-retinoid receptors X, each of which additionally has three subtypes (α, β, and γ). First and second generation retinoids can bind to several types of receptors, while third generation retinoids are characterized by higher receptor specificity. Topical retinoids for the treatment of acne (tretinoin, isotretinoin, and adapalene) affect the process of keratinization, accelerate exfoliation of dead cells, and reduce inflammatory changes. Tazarotene is used topically in the treatment of psoriasis, and bexarotene in the treatment of cutaneous T-cell lymphoma [90,91].

The following treatment of acne vulgaris is now used: (i) monotherapy with topical retinoid or benzoyl peroxide; or (ii) combination therapy with topical retinoid and benzoyl peroxide or antibiotic and benzoyl peroxide. Antibiotics used simultaneously (clindamycin, erythromycin) act by reducing *P. acnes* colonization in the skin, inhibiting inflammation, and relieving acne. Benzoyl peroxide, on the other hand, is used as a monotherapy or in combined therapy and has non-specific antimicrobial activity and limits the development of antibiotic-resistant *P. acnes*. It is also recommended when using a combination medication containing a retinoid (e.g., tretinoin) and an antibiotic (e.g., clindamycin). Several compound drugs are available for sale: benzoyl peroxide/adapalene, benzoyl peroxide/clindamycin, erythromycin/isotretinoin, and erythromycin/tretinoin. 

As a result of using retinoids, the thickness of the stratum corneum is reduced, which in turn leads to increased penetration of sunlight into the skin and faster sunburn. Due to the possibility of photosensitivity after the application of topical retinoids, it is recommended to avoid the sun and use sunscreen creams during increased sun exposure. These medicines should be used with caution in patients with previous photosensitivity symptoms. In the case of retinoids used externally in the form of creams, gels, and solutions, the therapy is usually started with preparations containing lower concentrations of active substances and then it is gradually changed by using preparations with higher concentrations. Drugs are administered once a day, usually in the evening, mainly due to the low photostability of the first-generation retinoids. Third-generation retinoids, due to modifications of their chemical structure, show greater lipophilicity and photostability, and they irritate the skin to a lesser extent compared to the first-generation retinoids.

Retinoids are a heterogeneous group of compounds in terms of photostability. Photochemistry of retinoids could proceed via, e.g., photoisomerization reactions, photooxygenation reactions, and photodegradation. The direction of transformation depends on many factors, including the concentration of the substance, drug formulation, exposure time, and type of radiation [92,93]. The photostability testing of retinoids reported in the literature is presented in Table 2.

Vitamin A refers to several substances with analogous structure that retain the activity of retinol. Retinol (all-trans retinol), as a precursor of retinoic acid, is a major regulator of the growth of epidermal cells and of their differentiation. Retinol is metabolized to active metabolites: all-*trans*-retinoic acid (retinyl palmitate, tretinoin) and 11-*cis*-retinal. Vitamin A absorbs ultraviolet radiation with an absorption maximum of λ = 325 nm. Gaspar et al. assessed the photostability of vitamin A in formulations containing chemical UV filters in two combinations differing in their photostability: photoinstable, including octyl methoxycinnamate, avobenzone and 4-methylbenzilidene camphor; and photostable, including octyl methoxycinnamate, benzophenone-3 and octocrylene [35]. The formulations were spread onto glass plates and exposed to UVA/UVB irradiation. The higher photostability of vitamin A was observed in both formulations containing UV filters in comparison to these not supplemented with UV filters [35].

Tretinoin (all-*trans* retinoic acid) and izotretinoin (13-*cis*-retinoic acid) are commonly used topical anti-acne agents. They are also used in the treatment of psoriasis and photodamaged skin. These two retinoids are very highly sensitive to light. Bassam et al. conducted research on the impact of UV solar simulated light, UVA, and visible light on the photostability of tretinoin and isotretinoin in ethanol and cream preparations [36]. Both tretinoin and isotretinoin underwent photoisomerization and photolysis following irradiation but tretinoin was more susceptible to degradation then isotretinoin. However, when comparing their photostability in cream formulations and ethanol, attention was paid to their greater stability in ethanol solutions. The authors of the articles explained this in terms of the diversity of components present in cream formulations which could interact with the investigated drugs by improving their photodegradation. In this context, UVA irradiation (as the major contributor to the photodegradation of tretinoin and isotretinoin) deserves special attention [36]. Irradiation within a wavelength range of 300–800 nm of ethanol solutions of tretinoin and isotretinoin leads to isomerization of tretinoin and isotretinoin to 13-*cis* and 9-*cis* isomers, respectively, within a few seconds of light exposure. Their incorporation into liposome complexes contributes to improved photostability [37]. The photostabilization of tretinoin by liposome incorporation has been the subject of numerous studies dealing with the photolability of this API and its formulation problems [38]. Brisaert et al. carried out accelerated stability analyses of dermatological preparations containing tretinoin including the influence of daylight and temperature only (25, 37, and 45 °C). The research revealed that the tretinoin degradation rate was severely affected by the presence of daylight at room temperature and at above the mentioned temperatures in all preparations (lotion and four hydrogels). 10% degradation of API was reached in the period of time from 1 to 181 h of irradiation depending on the formulation of preparation [39]. The studies of the influence of temperature showed that tretinoin was the most stable in the lotion, while the highest percentage of degradation was reported for Carbopol gel without Brij 35 S. It is clear that solubilizing agents have an impact on the stability of API. Therefore, the influence of solubilizing agents on the stability of tretinoin was also investigated. A comparison of the chemical stability of tretinoin in Carbopol gel with Brij 35 S and Carbopol gel without Brij 35 S indicates the negative influence of this solubilizing agent on the stability of tretinoin [39]. Furthermore, tretinoin in lotion underwent a fast photodegradation process in the daylight; on the other hand, the most stable were tretinoin gel preparations: Carbopol gel with and without Brij 35 S [39]. Brisaer et al. carried out investigations on tretinoin lotion to assess the stabilization effect of additives such as surfactants, cyclodextrins and proteins, dyes, and UVA and UVB filters under xenon lamp irradiation. According to the conducted research, tretinoin in lotion underwent 20% degradation within 30 min of irradiation and the addition of surfactant (Brij^®^s) or the use of β-cyclodextrin did not improve its photostability [40]. The results of the analysis in which tretinoin was irradiated with different radiation lengths were interesting. The most harmful wavelength was about 420 nm, which directly contributes to the photodegradation of tretinoin, not 350 nm—the wavelength of maximum absorption [40]. The combination of tretinoin with antibacterial agents, such as erythromycin, benzoyl peroxide, and clindamycin to improve the effectiveness of acne therapy is very common [41]. Martin et al. investigated the photostability of tretinoin when combined with benzoyl peroxide under visible light and UV radiation. The dark control showed the stability of tretinoin, but the presence of light, benzoyl peroxide, and light and benzoyl peroxide alone affected its stability under the same conditions. The presence of benzoyl peroxide or benzoyl peroxide and light resulted in degradation of about 80% and 95%, respectively, after 24 h [42]. An improvement of photostability was achieved after the use of tretinoin in micronized form. Comparison of photostability of micronized tretinoin in 0.05% gel with 0.025% gel with standard particle size after exposure to ultraviolet radiation and simulated sunlight for 8 h showed the greater photostability of micronized tretinoin. The degree of degradation was 11–12% in both cases of radiation for micronized tretinoin and 85–90% for tretinoin in the form of a conventional gel after applying fluorescent light and 84–89% after using simulated sunlight [43].

Lai et al. examined the impact of nanoemulsions and nanosuspensions on tretinoin photostability. The comparison of the photostability of tretinoin in methanol and tretinoin in nanoemulsion and nanosuspension was investigated using a 30 W lamp (366 nm). The samples were irradiated for 1 h. In the case of the methanolic solution, a residual concentration of 27% was present after irradiation. The use of nanoemulsion and nanosuspension improved the photostability of tretinoin. The concentrations of 83% and 52% of the initial amount of tretinoin were determined in nanosuspension and nanoemulsion, respectively. The half-life time was about 0.4, 0.9, and 3 h for the solution, nanoemulsion, and nanosuspension, respectively [44]. An isotretinoin micro-emulsion preparation has also been tested for the photostability under simulated sunlight conditions. Isotretinoin methanol solutions were a reference in which isotretinoin is completely photodegradable only a few minutes after exposure to UV radiation. The measured concentration of the tested substance after 240 min was 75% of the initial concentration in the micro-emulsion, while in the methanol solution it was completely degraded. Isotretinoin micro-emulsion preparation increased the half-life of the medicinal substance about 5-fold [45]. 

Adapalene [6-(3-(1-adamantyl)-4-methoxyphenyl)-2-naphthoic acid] is a naphthoic acid derivative with retinoid activity. Adapalene is a result of the search for a molecule more chemically stable than the leading topical retinoid tretinoin. This compound has all the benefits of the first-generation retinoids with minimization of the retinoid-associated skin irritation [94,95]. In this chemical entity, the chain with unstable double bonds is replaced by naphthoic acid. This change has caused an increase in stability during light exposure, improved resistance to oxidation by—e.g., benzoyl peroxide—and has decreased the irritating properties. Furthermore, the phenoxy adamantyl structure of adapalene has an impact on higher lipophilicity and thus on better skin penetration [94]. So far, the following impurities of adapalene have been described: impurity A (2,2′-binaphthalene-6,6′-dicarboxylic acid), impurity B (6[3(3hydroxytricyclo[3.3.1.1,37]dec1yl) 4methoxyphenyl] naphthalene-2-carboxylic acid), impurity C (1-(2-Methoxyphenyl)-tricyclo [3.3.1.13,7]decane), impurity D (1,1′-[4,4′-bis(methoxy)biphenyl-3,3′-diyl]bis(tri-cyclo[3.3.1.13,7]decane) [96].

Tolba et al. have developed a sensitive spectrofluorometric method to detect stability for the determination of adapalene, which was used during in vitro diffusion tests and in stability studies. Forced degradation studies involved alkaline and acidic degradation, oxidative degradation, daylight and UV light degradation at 254 and 366 nm for 12 h. Adapalene was stable under alkaline conditions (boiling with 2 M NaOH for 2 h), but was susceptible to acidic conditions. The total degradation of adapalene was observed after boiling with 1 M HCl for 10 min, whereas boiling with 0.3 M HCl for 10 min caused degradation of 28% of the parent drug. In terms of oxidative conditions the degradation was dependent on the concentration of the H_2_O_2_. A key finding of the stress degradation studies was the photolysis of adapalene after UVA (366 nm) and UVB (254 nm) irradiation for 12 h. The degradation proceeded via: (i) under acidic stress conditions breakage of adamantine group, (ii) during photolysis degradation of the naphthalene moiety into the corresponding 2-formyl cinnamaldehyde, and (iii) under oxidative conditions—the formation of a 1,4-naphthoquinone derivative [46].

The chemical stability of adapalene in combination with benzoyl peroxide was also investigated in the presence and in the absence of UV/VIS irradiation. In this case, a commercial formulation of adapalene 0.1% gel was mixed with an equal volume of a commercial formulation of benzoyl peroxide 10% lotion and irradiated over 24 h. Adapalene was stable under experimental conditions [42]. Roy et al. carried out forced degradation studies of adapalene in the presence of benzoyl peroxide in a topical pharmaceutical formulation. After 240 h of exposure to UV/VIS light or after 250 h of exposure to UV light, two main photoproducts were observed: benzoic acid (1.6%) and an unknown product (0.5%) at the relative retention time of about 0.93. The total percentage of the products was about 2.24% [47].

Tazarotene (ethyl 6-([4,4-dimethylthiochroman-6-yl]ethynyl)nicotinate) is used in the treatment of the most common form of psoriasis; namely, plaque psoriasis. This retinoid added to UVB phototherapy contributes to a significant reduction of the cumulative dose of UVB irradiation necessary to achieve at least 50% improvement in psoriasis compared to UVB alone or with UVB plus vehicle. [97]. Furthermore, the addition of tazarotene significantly enhances the efficacy of narrow-band UVB phototherapy [98]. Hecker et al. investigated the photostability of tazarotene gel while conducting in vivo research on the simultaneous use of tazarotene and UV light [48]. For this purpose, 2 g of 0.1% tazarotene gel was applied on the surface of 40 cm^2^ of the ventral aspect of the forearms, and then patients were exposed to UVB or UVA irradiation for 5 min depending on the recommendations. The research also took into account different degrees of psoriasis on the selected areas. After UV irradiation, the gel was collected and analyzed by an HPLC method. The concentration of tazaroten and its photoproducts (tazarotenic acid and AGN 190832) were determined. The percentages of degradation product AGN 190832 were 0.90, 1.05, and 0.01% for UVB, UVA, and control, respectively. The percentages of tazarotenic acid were even lower, 0.00, 0.05, and 0.01% for UVB, UVA, and control, respectively [48].

### 2.3. Antifungal Drugs

Antifungal drugs are characterized by a wide variety of chemical structures and a broad range of mechanisms of action. There are many antifungal drugs for both systemic and local use; however, the following groups play a special role in modern therapy of fungal infections: azole derivatives applied orally and topically, inhibitors of squalene epoxidase, morpholine derivatives, and polyene antifungals.

Azole derivatives have a broad spectrum of activities. The mechanism of action is based on fungistatic activity—by inhibiting the biosynthesis of ergosterol which is a component of the fungal cell membrane, and fungicidal activity—resulting from the change of the structure of the fungal cell membrane that is linked to the accumulation of azole drugs (for example, clotrimazole). However, despite their popularity, research into the photostability of azole antifungal drugs is very limited. The photodegradation of clotrimazole reaches 40% after 500 min of irradiation by polychromatic light according to the method which was developed by ECETOC. The investigated drug content was assessed by HPLC-UV, but the structure of photoproducts have not been described. The research was carried out in water to assess the fate of clotrimazole in the environment. The OSPAR Commission in its Background Document on Clotrimazole states that photolysis does not significantly contribute to the removal of this drug from the environment [49]. The assessment of the photostability of clotrimazole in methanol solution under UVA irradiation carried out by Kryczyk et al. showed no presence of photoproducts after 24 h. This irradiation was carried out in a KBF-ICH 240 APT.line™ climatic chamber (Binder GmbH, Tuttlingen, Germany) at 25 °C and 60% relative humidity using UVA radiation (320–400 nm) with a maximum emission at 365 nm. The presence of semiconductor photocatalysts TiO_2_ and ZnO under experimental conditions caused photocatalytic degradation of clotrimazole through the opening of the imidazole ring or loss of the imidazole moiety. An experiment carried out in phosphate buffer in the presence of the same photocatalysts showed the appearance of additional photocatalytic degradation products which were created as a result of hydroxylation of the phenyl rings [50]. Similar studies were conducted for bifonazole, but a different combination of TiO_2_ and/or ZnO was applied. Bifonazole was stable after UVA irradiation in the absence of catalysts and in all dark control samples. The photocatalytic degradation of bifonazole was the most efficient in the presence of both semiconductors. The determined values of kinetic parameters showed that the degradation process depends on the concentration of photocatalysts. Bifonazole photodegradation proceeded via hydroxylation of one of the phenyl rings or methanethiol groups, imidazole ring opening followed by further cyclization or loss of the imidazole moiety. The identification of photodegradation products was based on a UPLC/MS-MS analysis and, as a consequence, 10 photodegradation products of bifonazole were identified [51].

Among the triazole derivatives, we can distinguish itraconazole and fluconazole. The mechanism of action is based on inhibiting the enzyme responsible for the biosynthesis of ergosterol, which is a component of the fungal cell membrane resulting in increased permeability, inhibition of growth and fungicidal action. There are some research papers on itraconazole used as a topical dosage form [99,100]. Furthermore, it is registered as 1% gel (*w*/*w*) in India. Itraconazole has been found to be photo-unstable under UVA and UVB irradiation. The irradiation of itraconazole with UVB lamps (Hitachi, F15T8/BL, maximum output at ca 300 nm) was performed by Nardi et al. Three major photoproducts were formed in acetonitrile solutions, which were separated by preparative HPLC. The degradation of itraconazole proceeded via a dehalogenation process in ortho or para position. In the case of photoproduct 2 cyclization concerning the triazole moiety was also observed [52]. The photochemical properties of this drug are connected with its photosensitivity through generation of reactive aryl radicals as a result of the dehalogenation step [52]. Photocatalytic degradation of itraconazole in the presence of FeCl_3_, TiO_2_, and FeCl_3_/TiO_2_ is more efficient than its photolysis under UVA irradiation. The photodecomposition of itraconazole mainly includes a C-N bond cleavage step and the loss of one of the chloride atoms in the phenyl ring [53]. Kinetic evaluation of the photodegradation process has revealed that the activity of catalysts used decreases in the following order: FeCl_3_ > FeCl_3_/TiO_2_ > TiO_2_ [53].

Inhibitors of squalene epoxidase impair the synthesis of ergosterol by blocking the conversion of squalene to lanosterol. This group includes two antifungal agents applied topically to the skin: terbinafine and naphtyphine. Terbinafine is administered orally; however, due to the significant first pass metabolism and plasma protein binding (99%), topical administration of this drug is common and preferred [101,102]. Due to its high lipophilicity and keratophilicity terbinafine is concentrated in the stratum corneum, dermis, epidermis of the skin, and in the nails [103]. Photostability studies of terbinafine in the presence of selected UV filters and cytotoxicity studies of solutions after photocatalytic degradation using human skin fibroblast cells (BJ) ATCC™ were carried out by Kryczyk et al. [54]. The photodegradation process proceeded via oxidative deamination with the formation of 1-methylaminomethylnaphtalene or 1-naphthalenemethanol and loss of the side chain (*E*)-*N*,6,6-trimethyl-2-hepten-4-yn-1-amine. The formation of *Z*-terbinafine ((*Z*)-*N*,6,6-trimethyl-*N*-(naphthalen-1-ylmethyl)hept-2-en-4-yn-1-amine) is also possible [54].

The derivatives of morpholine include amorolfine, which is used only topically in the form of a cream and nail polish. The mechanism of its action is based on compromising the ability to ergosterol synthesis by blocking Δ14-reductase and then depositing false sterols in the cell membrane. No publications describing the stability of this widely used drug have been found.

Polyene antifungal drugs include amphotericin B and nystatin. Nystatin is a polyene antibiotic applied topically in the treatment of vaginal yeast infection as well as given orally in oral cavity infection. It is practically not absorbed through the skin or mucous membranes, nor is it absorbed from the gastrointestinal tract. In the case of amphotericin B, a clinical trial II has been performed to assess the safety and efficiency of topical application of amphotericin B cream (Anfoleish) in the treatment of cutaneous leishmaniasis [104]. Polyene antibiotics are photodegradable under UV irradiation. Amphotericin B and nystatin degrade after 2 h of irradiation. Photochemical instability applies not only to APIs but also to drug products [105]. The solubility and photostability of amphotericin B is improved by formatting of a complex with cyclodextrin, mainly γ-cyclodextrin [106].

### 2.4. Non-Steroidal Anti-Inflammatory Drugs (NSAIDs)

Non-steroidal anti-inflammatory drugs are among the most commonly used drugs in the pharmacotherapy of pain. NSAIDs inhibit prostaglandin synthesis by affecting the activity of cyclooxygenase (COX): constitutive COX-1 and induced COX-2 [107]. Baertschi et al. showed that 95 out of the 342 topical products authorized in the US which are listed in the USP should be stored in a light-protective packaging. In Europe, among topical products marked “protected from light” are, inter alia, piroxicam cream, and ibuprofen gel. Most of the NSAIDs are photoreactive; therefore, their photochemical properties have been investigated in detail. In addition, application of drugs from this group to the skin could be the source of drug-induced photosensitivity. Phototoxic and photoallergic reactions may appear as a result of both systemic or topical administration of NSAIDs, but higher concentrations in the skin after topical application of drugs lead to a higher frequency of photosensitivity for this route of administration [108].

Piroxicam is one of the NSAIDs which causes skin sensitivity to sun and therefore the photochemistry of this drug is a widely studied topic. Piroxicam belongs to the oxicam group of NSAIDs. It has a strong anti-inflammatory, as well as analgesic and antipyretic effect. The action of piroxicam is mainly based on the inhibition of cyclooxygenase (both COX-1 and COX-2). These enzymes participate in reactions that lead to the formation of prostaglandins from the lipids of cell membranes. Piroxicam is an oxicam derivative (4-hydroxy-2-methyl-*N*-(2-pyridyl) -2*H*-1,2-benzothiazine-3-carboxamide-1,1-dioxide), which differs structurally from other NSAIDs. The photodegradation of an ammonia solution (pH 11.8) containing three different concentrations of this compound (40 μg mL^−1^, 250 μg mL^−1^ and 2 mg mL^−1^) was concentration-dependent under irradiation by simulated sunlight for 480 min. Almost 100% of the initial amount of piroxicam was degraded after 288 min at a concentration of 40 μg mL^−1^ and after 480 min at a concentration of 250 μg mL^−1^. In the case of the highest concentration, 75.96% of the initial concentration underwent degradation [55]. Aminuddin et al. assessed the percentage of piroxicam photodegradation in buffer solution depending on the pH. The relationship between pH and the degradation rate is U-shaped, with an increase in the degradation rate in acidic and alkaline regions [109]. The inclusion complex of piroxicam with 2-hydroxypropyl-β-cyclodextrin increases the photostability by offering protection from daylight for up to 30 days [56]. Glass et al. identified four photodegradation products of piroxicam after irradiation of its methanolic solution: (i) 2-methyl-1,2-benzisothiazol-3(2*H*)-one1,1-dioxide; (ii) *N*-(2-pyridyl)-methoxy-formyl-amide as a result of oxygen incorporation into piroxicam; (iii) *N*-(2-pyridyl)-methoxyamide being a result of decarbonylation of *N*-(2-pyridyl)-methoxy-formyl-amide; and (iv) *N*-methyl-*N*′-(2-pyridyl)-ethane-diamide formed as a consequence of cleavage of the sulfur-nitrogen bond in the carboxylic acid. There was no statistically significant effect of 2-hydroxypropyl-β-cyclodextrin on the rate of piroxicam photodegradation [57].

The photostability of two well-known anti-inflammatory APIs was tested by Sammartino et al. After being dissolved in ultrapure water, diclofenac, and naproxen were irradiated using a light source that simulated sunlight (a mercury-vapor lamp coupled to a tungsten filament one) at 25 ± 1 °C. After the irradiation of the investigated samples for 90 h, an 88.4% and 91% decrease in the tested API concentrations for naproxen and diclofenac, respectively, was observed. The irradiance used during the experiment equaled 600 W/m^2^ which corresponds to the minimal irradiation during the sunniest hours during the day in Italy. Under the experimental conditions, the photodegradation rate was higher for diclofenac compared to naproxen in both dosage forms: solution and tablets [58]. The photodegradation process of diclofenac in aqueous solution was found to proceed via loss of one chlorine, then ring closure and chlorocarbazole acetic acid formation followed by the loss of the second chlorine and formation of hydroxycarbazole and its reduced product [57,62]. Drugs which cause photosensitivity commonly contain a chloroaromatic moiety. Diclofenac has been reported to be a photosensitive drug and the mechanism is based on free radical photodechlorination [57].

Solutions of diclofenac were also irradiated with a medium pressure 400 W mercury lamp in the presence and in the absence of cyclodextrins. The study of the impact of 2-hydroxypropyl-β-cyclodextrin on the photostability of diclofenac indicated that initially diclofenac in solution appeared to be more stable compared to the complex and after 30 min of irradiation the complex had higher photostability [57].

Naproxen was also found to undergo photolysis. The structures of naproxen photoproducts were determined using LC-ESI/MS by Hsu et al. [59]. Four degradation products were described in methanol solution after irradiation with a low-pressure quartz mercury lamp for 3 days: 1-(6-methoxy-naphthalen-2-yl)-ethanol, 1-(6-methoxy-naphthalen-2-yl)ethanone, methyl 2-(6-methoxy-naphthalen-2-yl)propanoate, and 2-ethyl-6-methoxynaphthalene [59]. Arany et al. additionally investigated the impact of UV (254 nm), VUV (172 nm), and UV/VUV (254/185 nm) on naproxen photolysis. Under experimental conditions, the photodecomposition depended on the applied irradiation decreasing in the following pattern: UV > VUV > UV/VUV [60]. The ecotoxicity of naproxen and its photodegradation products was assessed by Isidori et al. [61]. An assay performed on the rotifer *B. calyciflorus*, crustaceans: *C. dubia* and *T. platyurus*, and the alga *P. subcapitata* showed that photoproducts were more toxic than naproxen. Genotoxicity tests did not show genotoxic and mutagenic effects of degradation products [61].

Topical application of naproxen or ketoprofen can result in the phototoxic and photoallergic reaction after UVA irradiation. Ketoprofen is the most common cause of the photosensitivity induced by NSAIDs. UV photolysis of ketoprofen was carried out with a low pressure (LP) Hg lamp (λ = 254 nm) and the applied exposure time corresponded to 0, 100, 500, 750, 1000, and 1500 m·J·cm^−2^ [110]. Photolysis with a UV Hg lamp, medium pressure (MP), was carried out using a mixture of compounds: ketoprofen, diclofenac, and atenolol in pure water. The experiment allowed the proposing of phototransformation pathways for photolysis and identification of the degradation products of the investigated compounds. The major products of ketoprofen in LP and MP photolysis are 2-(3-(carboxyoxomethyl)phenyl)propanoic acid and 2-(3-(carboxy(hydroxy)-methyl)phenyl) propanoic acid arising as a result of oxidative ring opening [110]. The UV (254 nm) photolysis of ketoprofen and ibuprofen was also investigated by Szabo et al. [63]. After 90 s of irradiation, ketoprofen was completely degraded. Four photoproducts were identified i.a. 3-hydroxyethyl benzophenone which had been described earlier by Matamoros et al. [63,64]. Under UV irradiation, ketoprofen underwent degradation, which in vitro in the presence of rat hepatocytes, fibroblasts, or red blood cells was connected with the formation of radical intermediates and damage to the cell membrane, as well as membrane lipids peroxidation and red blood cell hemolysis [65,66]. Furthermore, ketoprofen may cause the induction of DNA damage and formation of pyrimidine dimers [108]. Atarashi et al. revealed that the addition of butyl methoxydibenzoylmethane into a topical formulation containing ketoprofen reduced the photoallergic reaction caused by this drug. Butyl methoxydibenzoylmethane is a UVA filter, but unlike octocrylene and benzophenone-3 shows no cross-reactivity with ketoprofen [111]. It should be emphasized that the benzophenone moiety in ketoprofen plays a key role in its photosensitivity reactions. Hence, patients with a photoallergic reaction to ketoprofen should avoid sunscreen containing octocrylene and benzophenone-3.

Ibuprofen is a traditional NSAID commonly used for its analgesic and anti-inflammatory properties. Ibuprofen is a non-selective, reversible inhibitor of COX-1 and COX-2. It is considered to be relatively stable but, in aqueous solution, it undergoes direct photolysis and self-sensitization which is based on photo-oxidation. The major photoproducts of ibuprofen have been identified as 1-(4-isobutylphenyl) ethanol and 4-isobutylacetophenone [67]. Furthermore, the generated photoproducts of ibuprofen are more toxic than the parent drug [67,68]. 4-isobutylacetophenone, which can be formed in the environment during direct photolysis and reactions with OH^•^ and with the triplet states of chromophoric dissolved organic matter was toxic to cell membranes, causes protein dysfunctions and protein stress, and affects the nervous system [69,70,71].

### 2.5. UV Filters

Efficient sun protection could be provided by synergistic action of various combinations of inorganic and organic UV-filters. Physical filters such as TiO_2_ and ZnO reflect and scatter UV radiation. These are filters showing a wide spectrum of protection against both UVA and UVB. ZnO is more effective in the UVA range, while TiO_2_ better protects against UVB and short-wave UVA, which is why they are often used together. They are stable, but leave a white layer on the skin, which is why they are currently used in micronized form. Chemical filters are organic compounds that have a high molar absorption coefficient in the UV range (100–400 nm). Chemical solar filters work by absorbing radiation due to the presence of numerous unsaturated bonds and moieties with free electron pairs. Absorption can lead to photochemical reactions in these molecules, such as *trans-cis* transformation, or keto-enol tautomerism. Chemical filters can be divided into several groups: p-aminobenzoic acid derivatives—4-aminobenzoic acid (PABA), benzophenone derivatives—benzofenone-3 and sulisobenzone, salicylic acid derivatives—homomenthyl salicylate (homosalate), cinnamic acid derivatives—octyl methoxycinnamate (OMC), camphor derivatives—4-methylbenzylidene camphor (4-MBC), triazine derivatives—bis-ethylhexyloxyphenol methoxyphenyl triazine (Tinosorb S), methylene bis-benzotriazolyl tetramethylbutylphenol (Tinosorb M), and others. Another division was made due to protection in the specified UV range—PABA and OMC, homosalate, 4-MBC protecting in the UVB range; butyl methoxydibenzoylmethane (avobenzone), benzofenone-3, Mexoryl SX and Mexoryl XL protecting in the UVA range; and Tinosorb S, Tinosorb M, and octocrylene protecting in the UVA and UVB range. Parsol SLX is an oligomer belonging to the new generation of filters protecting against UVB radiation with a maximum absorption of 310 nm (Polysilicone-15).

The photoinstability of filters could result in a change to their photoprotective proprieties and safety profiles. Organic UV filters after absorption of UV radiation may lose their excitation energy through, e.g., chemical transformation. In the case of reversible transformations, the system is stable, unlike non-reversible transformation where photodegradation occurs. The capability to dissipate excitation energy via reversible transformations, e.g., E/Z isomerization, is a desirable process for UV filters, but requires evaluation of absorption curves in terms of their shape and the magnitude of extinction coefficients for mixtures of E/Z isomers. The E ⇄ Z fast and reversible isomerization of benzylidene camphor derivatives, e.g., 4-MBC, when irradiated by UV is commonly known [72]. Absorption of UVA irradiation could lead to *trans-cis* isomerization, which is in accordance with the assumption that the most probable photochemical reaction for derivatives of cinnamic acid is *trans-cis* photoisomerization [73]. Broadbent et al. likewise defined one degradation product as a result of photostability studies (UV irradiation at wavelengths 313 nm) of ethylhexyl methoxycinnamate (*trans*-EHMC). *Cis*-2-ethylhexyl-*p*-methoxycinnamate is a product that results from the conversion of *trans*-EHMC. Furthermore, *trans*-EHMC irradiated at wavelengths above 300 nm with more intense source has undergone photodimerization via a cycloaddition reaction [74]. Therefore, one of the most important characteristics of organic UV filters is photostability. Chemical filters can absorb the radiation, which can cause chromophore destruction and thus lead to a decrease or even loss of its ability to absorb energy through photoisomerization processes (avobenzone, OMC) [75,78,79] fragmentation, and generation of free radicals [112] or photoaddition [76]. These are not reactions based on the ‘all or nothing’ response, but there is a gradual loss of filter protective properties over time, and the resulting photoproducts and free radicals with unknown properties can react with other cream ingredients, skin, and sebum, which may cause phototoxic, photoallergic, and other toxicological effects that are difficult to predict. High photostability of the mentioned chemical UV filters is an important and desirable requirement to achieve the effectiveness of sunscreen products. In recent years, the use of avobenzone and other UVA filters has increased significantly due to the proven harmfulness of this radiation. Research is being conducted into the stability of already use filters not only in the context of determining the structure of degradation products, and kinetic parameters of the degradation process but also in the context of safety of use in both in vitro and in vivo tests [35,113,114]. There are increasing numbers of reports about the impact of UV radiation on the photodegradation of specific chemical filters and their combinations providing full protection in both UVB and UVA. It turns out that some combinations are very unfavorable for the photostability of UV absorbers, while others improve the initial stability of UV filters [115]. The best known example is avobenzone, which is the most commonly used chemical filter due to its strong protection in the range from 310 to 400 nm with a maximum at 360 nm [77]. However, it has been pointed out that avobenzone requires the presence of UVB filters—e.g., octocrylene or 4-MBC—then these substances prevent its degradation, which leads to strengthening of its protective capabilities and stability. That is why modern preparations contain both UVB and UVA filters to ensure a full spectrum of protection.

Avobenzone is 50–90% photodegradable after one hour of exposure to UV radiation, and should not be combined with any of the most commonly used UVB *trans-EHMC* and OMC filters because as a result of the reaction between them a new compound is formed, which leads to loss of UVA and UVB protection [76,77]. The commonly used OMC also undergoes degradation. Tinosorb S prevents the photodegradation of avobenzone, but also acts photoprotectively in creams simultaneously containing avobenzone and OMC [76].

In recent years, the problem of the photostability of drugs during administration appears to be increasingly emphasized and discussed [17,21]. This is in particular an issue for drugs used in topical preparations because their application on external body surfaces causes a high probability of exposure to UVR. Additionally, there is a high probability of interaction of topical pharmaceutical products used concomitantly with cosmetics. It should be pointed out that cosmetics might contain different ingredients such as inorganic UV filters (nanoparticles of ZnO or TiO_2_) which may show a high photocatalytic activity. Organic UV absorbers could be used in the photostabilization process of photolabile drugs. This strategy has been used in the case of diclofenac and ketoprofen, where stabilization was achieved by use of EHMC or ethylhexyl salicylate [116]. In contrast, light energy equal to or higher than a bandgap of TiO_2_ or ZnO (*λ* < 380 nm) generated an electron in a conductive band (e_CB_^−^) and a positive hole in the valence band (h_νB_^+^) pair. The holes in the valence band, as strong oxidizing agents, could generate hydroxyl radicals (OH^•^). Furthermore, the electrons in conduction band reduce oxygen to $O_{2}^{•}$ radicals. Additional reaction can lead to the formation of hydrogen peroxide and OH^•^. The reactive oxidizing species could lead to oxidation of the organic compounds [117,118,119,120].

(1) $${\mathrm{TiO}}_{2}+h\nu \;\left(<380\;\mathrm{nm}\right)\to e_{\mathrm{CB}}^{-}+h_{\nu B}^{+}$$

(2) $$\mathrm{ZnO}+h\nu \;\left(<380\;\mathrm{nm}\right)\to e_{\mathrm{CB}}^{-}+h_{\nu B}^{+}$$

(3) H2O+hνB+→O•H+H+

(4) $$e_{\mathrm{CB}}^{-}+O_{2}\to O_{2}^{•-}$$

(5) $$O_{2}+2H^{+}+e_{\mathrm{CB}}^{-}\to H_{2}O_{2}$$

(6) H2O2+eCB−→O•H+OH−

(7) OH−+hνB+→O•H

(8) H++O2•−→H•O2

## 3. Photostabilization Strategies of Selected Dermatological Drugs

Dermatological preparations are mainly semisolid dosage forms applied to the skin. Different methods are required to test their photostability than for solid or liquid preparations. Different methods of photostabilization are also recommended. Furthermore, it is necessary to verify the stability of the API in the tested dosage form, as a substance stable in solution may turn out to be unstable in, e.g., cream. In this context, research dealing with the photodegradation of dermatological drugs as well as photostabilization strategies is summarized in Table 3. The main factors playing an important role in overcoming photoinstability are: formulation factors, UV-absorbers and pigments, antioxidants, cyclodextrins inclusion, vesicular systems, and combination of different techniques [20,21,38,84].

## 4. Drug-Induced Photosensitivity

Initially, increased interest in sunscreens was related to the existing relationship between exposure to UV radiation and skin aging and pigmentation disorders; that is, aesthetic considerations. It is now well known that protection against UV radiation is extremely important for health reasons. Adverse effects of UV radiation include burns, photodermatoses (polymorphous light eruption, urticaria), and photoallergic and phototoxic reactions. Furthermore, light sensitive substances applied to the skin, e.g., perfumes, essential oils, drugs, or molecules supplied to the skin by the circulatory system (e.g., hypericin or selected cardiovascular drugs and antibiotics), might induce photosensitivity. Drug-induced photosensitivity is associated with the presence of two agents: light (ultraviolet or visible radiation) and drug (systemically or locally administrated). Photosensitivity reactions are classified as phototoxic reactions or photoallergic reactions, which are often difficult to distinguish. UVA radiation (320–400 nm) is much more often responsible for inducing hypersensitivity reactions to light because of deeper penetration into the skin. Many drugs—e.g., retinoids, salicylates, cinnamates, benzophenones, itraconazole, and voriconazole—are responsible for the photosensitivity reactions. Table 4 contains selected drug classes that have been reported to induce photosensitivity reactions. Phototoxicity is an acute reaction caused by damage initiated by the light-induced degradation of photoreactive or photoactive molecules. Because of the presence of chromophores in a drug’s structure, their molecules absorb high-energy UV radiation which results in molecular changes or generates reactive oxygen species [122,123]. The active pharmaceutical substance or its metabolite must be present in the skin tissue during UV irradiation. Depending on the type of reaction with endogenous molecules following the energy absorption by drug molecules, mechanisms of phototoxicity are categorized into two modes of action: direct, when a drug directly reacts with the endogenous molecules; and indirect, when photoproducts react with endogenous molecules [124]. Symptoms include skin irritation, erythema, pruritus, and edema, which are similar to those with excessive sunburn only in regions exposed to the sun. Photoallergic reactions are rare, independent of dose, and appear after a few days of exposure to radiation. They are associated with the immune cell response, the drug or its degradation products act as haptens, via antigen presenting cells or T lymphocytes, which triggers an allergic reaction on repeated exposure [122,123,124,125]. Currently, the most appropriate test for the phototoxicity testing of soluble compounds is OECD TG 432 (in vitro 3T3 NRU phototoxicity test) [126]. Phototoxicity is assessed on the basis of the average cell viability (base cell-BALB/c 3T3 cell—mouse fibroblast) in the presence of the tested compound under UV/VIS irradiation or without it [124].

## 5. Conclusions

Photodegradation of APIs may occur during a drug’s production process; its packaging, warehousing, or storage; as well as its correct use by patients. The photodegradation process of drugs poses a risk due to possible loss of API and formation of by-products with unknown effect. The assessment of a drug’s photostability requires a comprehensive approach. There are many factors to be considered such as the concentration of API, intensity and wavelength of irradiation, pH, polarity of solvents, and excipients. The overall objective of the review was to collate knowledge on the photostability of drugs applied to the skin, as well as factors potentially affecting this process.

## Figures and Tables

**Table 1:** Selected classes of dermatological drugs described in terms of the photostability.

Drug Class	Active Pharmaceutical Ingredient	Ref.
Glucocorticosteroids	pregna-1,4-dien-3,20-diones	[24]
	betamethasone and its esters	[25,26]
	betamethasone-17 valerate	[27]
	mometasone furoate	[28]
	hydrocortisone 21-acetate	[29]
	prednisolone	[30]
	fluocinolone 16,17-acetonide	[31,32]
	desonide	[33,34]
Retinoids	vitamin A	[35]
	tretinoin	[36,37,38,39,40,41,42,43,44]
	isotretinoin	[36,37,45]
	adapalene	[46,47]
	tazarotene	[48]
Antifungal drugs	clotrimazole	[49,50]
	bifonazole	[51]
	itraconazole	[52,53]
	terbinafine	[54]
Non-steroidal anti-inflammatory drugs	piroxicam	[55,56,57]
	naproxen	[58,59,60,61]
	diclofenac	[57,58,62]
	ketoprofen	[63,64,65,66]
	ibuprofen	[67,68,69,70,71]
UV filters	4-methylbenzylidene camphor	[72]
	octyl methoxycinnamate	[73,74,75]
	avobenzone	[76,77,78,79]

**Table 2:** Photostability of retinoids reported in the literature.

Retinoid	Presentation of Samples	Light Source	Irradiation Time/Dose	Ref.
Adapalene	- ethanol solution - 25 mL volumetric flasks	CAMAG UV-lamp, S/N 29000, dual wavelength 254/366 nm (Switzerland)	day light, UV-light 254 nm UV-light 366 nm irradiation time—12 h distance—15 cm	[46]
Adapalene with benzoyl peroxide	- adapalene 0.1% gel and 10% benzoyl peroxide lotion - 10 mL plastic syringes	monochromatic sodium lamp type NA 55 W (Osram), and fluorescent lighting tubes for normal room lighting	inactinic light actinic light 24 h	[42]
Adapalene	- gel	photostability chamber (SUN TEST XLS+, Atlas, USA).	visible light for 240 h (1.2 million lux h), UV light for 250 h (200 W h/m^2^) temp. 25 °C	[47]
Tazarotene	- gel 0.1% - 2 g applied on 40 cm^2^ area on the ventral aspect of the forearms	UVB—Light Sources FS72 T12-UVB-HO bulbs UVA—FS72 T12-BL HO/50R bulbs covered with filters blocking UVB and lower wavelengths.	phototherapy UVB 100 to 150 mJ/cm^2^ UVA 15 to 22 J/cm^2^	[48]
Tretinoin with benzoyl peroxide	- tretinoin 0.025% gel and 10% benzoyl peroxide lotion - 10 mL plastic syringes	monochromatic sodium lamp type NA 55 W (Osram), and fluorescent lighting tubes for normal room lighting	inactinic light actinic light 24 h	[42]
Tretinoin	- lotion 0.05% (*w/v*) tretinoin - quartz cuvette	XBO 450 W high pressure xenon lamp	distance of 28 cm temperature in the cuvette never exceeded 36 °C	[40]
Tretinoin	- RA-liposomes - ethanol solution - 1 cm quartz cuvette	light testing cabinet Suntest CPS+ (Heraeus, Milan, Italy), equipped with a Xenon lamp	light dose of 21 kJ min^−1^ m^−2^, temperature of 25 °C. 0.5–240 min	[37]
Tretinoin	- tretinoin (0.025%) cream spread uniformly over the cover of a 35 mm tissue culture dish - ethanol solution (0.025%) in Eppendorf centrifuge tubes	solar simulator, model 91293, (Oriel Corporation, Stratford, CT, USA) equipped with 1000 W Xenon lamp Luzchem expo Panels composed of 5 Sylvania 8 W cool white light tubes	distance 20 cm at 365 nm from the source, the SSL dose was 7.63 mJ/cm^2^/sec UVA and 0.40 mJ/cm^2^/sec UVB radiation, UVB/UVC blocking filter the dose at 365 nm from the source was 5.39 mJ/cm^2^/sec UVA radiation with residual UVB dose of 3.16 µJ/cm^2^/sec.	[36]
Isotretinoin	- 13RA-liposomes ethanol solution - 1 cm quartz cuvette	light testing cabinet Suntest CPS+ (Heraeus), equipped with a Xenon lamp Luzchem expo Panels composed of 5 Sylvania 8-W cool white light tubes	light dose of 21 kJ min^−1^ m^−2^, temperature of 25 °C. 0.5–240 min	[37]
Isotretinoin	- isotretinoin (0.025%) cream spread uniformly over the cover of a 35 mm tissue culture dish - ethanol solution (0.025%) in Eppendorf centrifuge tubes	solar simulator, model 91293, (Oriel Corporation) equipped with 1000 W Xenon lamp	distance 20 cm at 365 nm from the source, the SSL dose was 7.63 mJ/cm^2^/sec UVA and 0.40 mJ/cm^2^/sec UVB radiation; distance 20 cm at 365 nm from the source, the dose was 5.39 mJ/cm^2^/sec UVA radiation with residual UVB dose of 3.16 µJ/cm^2^/sec (UVB and UVC blocking filter)	[36]
Vitamin A	- formulation spread onto an area of 10 cm^2^ (approximately 4 mg/cm^2^) of a glass plate	96000 Oriel 150 W Xenon arc solar simulator (Oriel Corporation)	UVA/UVB irradiation (280–400 nm) UVB dose of approximately 334.8 mJ/cm^2^ 30 min	[35]

**Table 3:** Strategies for improving the photostability of selected dermatological drugs.

Active Pharmaceutical Ingredients	Photostabilizers/Excipients	Form	Percent Loss	Irradiation Dose/Time/Type/Source	Ref
Betamethasone valerate	control	cream	49.2 ± 0.92	UV lamp (300 W, Ultra-Vitalux Osram) 300–400 nm, the intensity of light-16 000 lx, up to 2 h of irradiation	[27]
	titanium dioxide (light scattering)		17.78 ± 1.24		
	vanillin (radical scavenger)		27.6 ± 1.36		
	butyl hydroxytoluene (radical scavenger)		31.0 ± 1.22		
Betamethasone valerate	control	gel	42.5 ± 1.64	UV lamp (300–400 nm) the intensity of light-16 000 lx	[27]
	titanium dioxide (light scattering)		7.2 ± 0.98		
	vanillin (radical scavenger)		13.8 ± 1.44		
	butyl hydroxytoluene (radical scavenger)		21.9 ± 1.60		
Betamethasone valerate cream	control (without the preservative)	topical ointment 0.1%	about 30%	UVB (5 J/cm^2^)– Philips PL-S 9W/12 lamp mainly emitting at 312 nm	[25]
	chlorocresol (excipient-preservative)		less than 10%		
Hydrocortisone 21-acetate	control (without the preservatives)	commercial formulation (cream)	40%	UVB (15 J/cm^2^)– Philips PL-S 9W/12 lamp mainly emitting at 312 nm	[29]
	parabens: methyl- and propyl p-hydroxybenzoates (excipients—preservatives)		20%		
Triamcinolone acetonide	control (without the preservatives)	basis cream DAC	38%	3 h of irradiation, Suntest CPS+, 415 W/m^2^	[84]
	pigmented creams (ZnO, TiO_2_)		95%		
Desonide	control	hair solution (0.1%)	61%	UVA irradiation (1350 W h/m^2^) 15 h of irradiation	[34]
	benzophenone-3 (UV-filter, 0.3%)		1.49%		
Vitamin A	control: 0.6% (*w*/*w*) vitamin A palmitate (1,700,000 UI/g)	topical formulation	n.d.	30 min UVA/UVB irradiation (280–400 nm) 96000 Oriel 150 W xenon arc solar simulator (Oriel Corporation), 0.186 mW/cm^2^, UVB dose 334.8 mJ/cm^2^	[35]
	octyl methoxycinnamate, avobenzone, 4-methylbenzilidene camphor		enhanced vitamin A stability		
	octyl methoxycinnamate, benzophenone-3, octocrylene		enhanced vitamin A stability		
Tretinoin	solution	ethanolic solution	92%	Sunset CPS+ (Heraeus)-xenon lamp (300–800 nm) 250 W/m^2^ for 240 min	[37]
	liposomes	liposomes	40%		
Tretinoin	micronized tretinoin (0.05%)	gel	9%	UVA light (315–400 nm) 22 W/m^2^	[43]
	tretinoin (0.025%)	gel	72%		
Tretinoin	control:	Methanolic solution	63%	1 h of irradiation, 30 W lamp-366 nm (Min UVIS, Desaga, GmbH, Germany)	[44]
	Nanosuspension tretinoin (0.035%)	nanosuspension	17%		
	Nanoemulsion tretinoin (0.035%)	nanoemulsion	48%		
Tretionoin	control:	methanolic solution	incorporation in vesicles always improved the photostability of tretinoin	UV lamp set at 366 nm (Min UVIS, Desaga, GmbH) fluorescent light	[121]
	niosomes tretinoin	vesicular suspensions			
Isotretinoin	control	methanol solution	84%	natural sunlight (>20,000 Lux)	[45]
	micro-emulsion tretinoin (0.035%)	micro-emulsion formulation	25%		
Diclofenac	control	solution	the drug appears to be more stable than the complex for T < 30 min and thereafter degrades rapidly (the complex is more stable)	400 W mercury lamp	[57]
	2-hydroxypropyl-β-cyclodextrin	cyclodextrin			
Piroxicam	control	piroxicam	not affected the rate of photodegradation	n.d.	[57]
	2-hydroxypropyl-β-cyclodextrin	piroxicam-β-cyclodextrin			
Piroxicam	control	piroxicam	complex improved photostability	daylight up to 30 days	[56]
	2-hydroxypropyl-β-cyclodextrin	piroxicam:2-hydroxypropyl-β-cyclodextrin complex			
Avobenzone	control	prepared formulation	56%–70% (different concentration of avobenzone)	optically filtered xenon arc source (Multiport Solar UV simulator, Solar light, Philadelphia, PA, USA) UV irradiance adjusted at 1 mean effective dose [MED]/min	[76]
	tinosorb S	formulation with tinosorb S	5%–15%		

n.d.—not defined.

**Table 4:** Photosensitizing drugs used in dermatology.

Class of Drug	Active Pharmaceutical Ingredient	Photosensitivity	Action Spectra	Ref.
NSAID	Ketoprofen	Phototoxic reaction Photoallergic reaction	UVA	[108,127,128]
	Naproxen	Phototoxic reaction Photoallergic reaction	UVA	[129]
	Piroxicam	Photoallergic reaction	UVA	[130,131]
	Ibuprofen	Phototoxic reaction	UVA	[132]
Antifungal agents	Itraconazole	Phototoxic reaction Photoallergic reaction	Unknown	[123,133]
	Voriconazole	Phototoxic reaction	UVA	[123,134,135,136]
	Ketoconazole	Phototoxic reaction	Unknown	[123,137]
	Griseofulvin	Phototoxic reaction	UVA	[123,138]
Retinoids	Etretinate/the major metabolite of etretinate	Phototoxic reaction	UVA/poss. UVB	[139,140]
	Isotretinoin	Phototoxic reaction	UVA/poss. UVB	[140,141]
	Tretinoin	Phototoxic reaction	Unknown	[140]
UV filters	PABA derivatives	Photoallergic reaction	UVA	[142]
	Benzophenones	Photoallergic reaction	UVA	[142]
	Isopropyl dibenzoylmethane	Photoallergic reaction	UVA	[142]
	Cinnamates	Photoallergic reaction	UVA	[143]
	Camphor derivatives	Photoallergic reaction	UVA	[142]
	Avobenzone	Photoallergic reaction	UVA	[142]

poss.—possibly.
